# P-1233. Application of Pharmacometrics in the Personalized Colistin Dosing Recommendation

**DOI:** 10.1093/ofid/ofae631.1415

**Published:** 2025-01-29

**Authors:** Hyeri Seok, Suein Choi, Seunghoon Han, Won Suk Choi, Dae Won Park

**Affiliations:** Korea University Medicine, Ansan, Kyonggi-do, Republic of Korea; Catholic University of Korea, seoul, Seoul-t'ukpyolsi, Republic of Korea; Catholic University of Korea, seoul, Seoul-t'ukpyolsi, Republic of Korea; Korea University Ansan Hospital, Ansansi, Kyonggi-do, Republic of Korea; Korea University Ansan Hospital, Ansansi, Kyonggi-do, Republic of Korea

## Abstract

**Background:**

Colistin is frequently used as a treatment for infections caused by carbapenem-resistant Gram-negative bacteria. Colistin dosing should be adjusted according to the patient's renal function because the prodrug, colistin methanesulfonate (CMS), is partially removed by the kidney. Intensive care unit (ICU) patients may require individualized dosing to prevent nephrotoxicity or treatment failure due to the highly variable pharmacokinetics (PK) of colistin.

**Figure 1.**
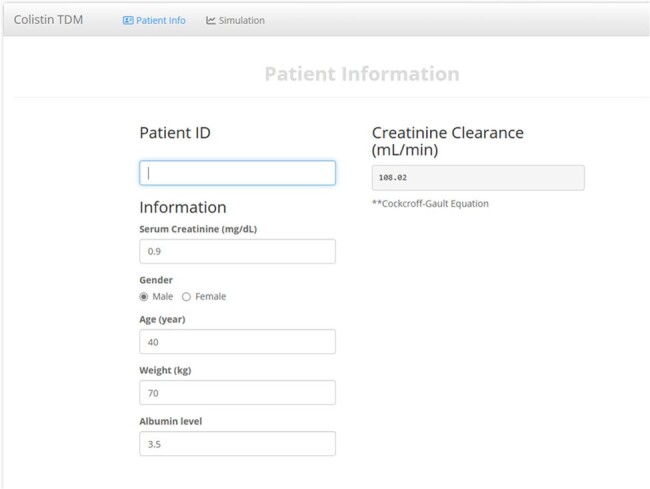
Example of colistin dosing recommendation program – Patient information input

**Methods:**

Colistin plasma concentration data from ICU patients who received colistin from June 2019 to August 2022 in Korea University Hospital were included in the dataset for this population PK analysis. We developed a population PK model for colistin using ICU patients, and established therapeutic drug monitoring software. A population PK analysis was performed using the first-order conditional estimation method with interaction in non-linear mixed effects modeling. We used the open-source R Shiny program to establish Colistin TDM software. The structural PK model and the final estimates of both fixed-effect and random-effect parameters were translated into an R code.

**Figure 2.**
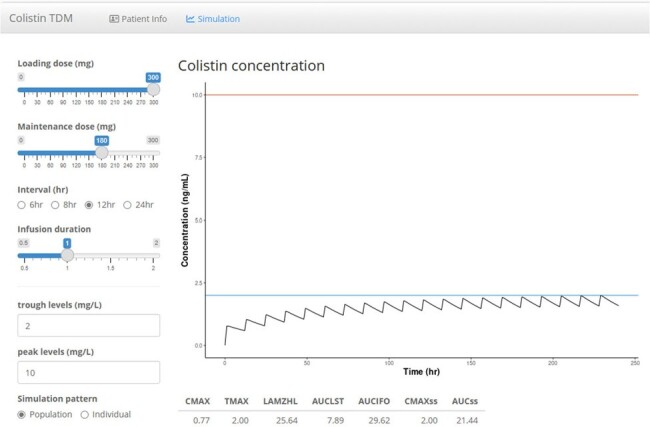
Example of colistin dosing recommendation program – Simulation output Recommendations provide the loading and maintenance doses based on patient information. Maintenance dose depends on the predicted colistin trough and peak levels, and the interval and infusion time of colistin administration.

**Results:**

We enrolled 176 colistin observations from 22 patients in the dataset. A two-compartment, first-order elimination model best described the PK of Colistin A, one-compartment, first-order elimination model for PK of Colistin B, and one-compartment, first-order elimination model for PK of CMS. In the results from the covariate analysis, GFR estimated by Cockcroft–Gault equation and albumin level was included as a statistically significant covariate of total clearance of CMS, and body weight was selected as a covariate of CMS volume of distribution. The two-compartment structure model for Colistin and CMS, the final estimates of both fixed effects and random effects from the PK model and the log-likelihood Equation (5) for estimating ηi were implemented in R script. A draft version of the software is now available at http://mychloe00.shinyapps.io/colistin_new.

**Conclusion:**

We developed colistin TDM software using PK parameters in ICU patients for individualized colistin administration. Further research is needed to determine whether colistin is actually administered within the therapeutic range through the application of this software.

**Disclosures:**

**All Authors**: No reported disclosures

